# Gastrointestinal stromal tumour and hypoglycemia in a Fjord pony: Case report

**DOI:** 10.1186/1751-0147-50-9

**Published:** 2008-05-16

**Authors:** Henning A Haga, Bjørnar Ytrehus, Inger J Rudshaug, Nina Ottesen

**Affiliations:** 1 Department of Companion Animal Clinical Science, Norwegian School of Veterinary Science, P.O. box 8146 Dep., 0033 Oslo, Norway; 2 Department of Basic Sciences and Aquatic Medicine, Norwegian School of Veterinary Science, P.O. box 8146 Dep., 0033 Oslo, Norway; 3 Section for Wildlife Diseases, National Veterinary Institute, Oslo, Norway

## Abstract

**Background:**

Neoplasia may cause hypoglycemia in different species including the horse, but hypoglycemia has not previously been reported in the horse associated with gastrointestinal stromal tumours.

**Case presentation:**

A case of a gastrointestinal stromal tumour in a Fjord pony with severe recurrent hypoglycemia is presented. The mechanism causing the hypoglycemia was not established.

**Conclusion:**

This case indicates that a gastrointestinal stromal tumour may cause hypoglycemia also in the horse.

## Background

Clinical signs of hypoglycemia in adult equines are unusal. In humans and canines hypoglycemia caused by neoplasia is well established [1,2], a few reports have described this occurrence in the horse [3-7]. Hypoglycemia has not previously been described in association with gastrointestinal stromal tumours in this species. This report describes a horse with a gastrointestinal stromal tumour and hypoglycemia.

## Case presentation

### History

A 12-year old, 420 kg Fjord pony stallion was referred to the equine clinic at the Norwegian School of Veterinary Science for evaluation of intermittent colic, and episodes of collapse. During the 3 weeks prior to admission the horse had a history of several episodes of ataxia, apparent blindness, headshaking, profuse sweating at the hindquarters, collapse and clinical signs of colic. The horse recovered spontaneously from these episodes, but on two occasions the horse was treated with flunixine meglumine intravenously and mineral oil per os and then recovered. The day prior to admission, the referring veterinarian had identified a firm mass cranio-ventrally in the abdomen by per rectum abdominal palpation.

### Clinical presentation

On initial examination at the Norwegian School of Veterinary Science the horse was in poor body condition with general muscle wasting and had a potbellied appearance. He was also sweating and appeared mildly depressed. Body temperature was 38.2°C, heart rate 48 beats/min, respiratory rate 36 breaths/min and the oral mucosa was brick coloured with a capillary refill time of 2–3 seconds. The horse passed normal faeces and urinated. At per rectum abdominal palpation a large mass ventrally in the abdomen was identified. An arterial blood sample was obtained and analysed within 10 minutes, which identified hypernatremia (sodium; 141 mmol/L; reference range, 135–139 mmol/L), hyperchloremia (chloride 107 mmol/L; reference range, 92–102 mmol/l), hypoglycemia (glucose 3.8 mmol/L; reference range, 4.6–7.3 mmol/L).

An impaction of the large colon was suspected and 2 L mineral oil (SPC68WOM, Keddel & Bommerts, Oslo, Norway) and 300 g sodium sulphate (Norsk Medisinaldepot AS, Oslo, Norway) in 4 L of water was administered by nasogastric intubation. In addition intravenous infusion of 10 L Ringers acetate (Fresenius Kabi AB, Uppsala, Sweden) and 4 L 5% glucose solution (Fresenius Kabi AB, Uppsala, Sweden) was initiated and was ended within 8 hours. Flunixine meglumine 0.25 mg/kg IV (Finadyne, Schering-Plough Animal Health, USA) was given every 8 hours. During the following 8 hours, the horse improved clinically, pulse and respiratory rates fell to 36 beats/min and 10 breaths/min respectively and body temperature decreased to 37.8°C. Two hours later, the horse clinically deteriorated, pulse and respiratory rate were 48 beats/min and 16 breaths/min respectively. The horse became ataxic, had focal muscle spasms in the head region and fell over several times until it was unable to get up. Ten minutes later the horse rose with difficulty, but appeared depressed. The following eight hours the horse stood quietly, uninterested in the surroundings, had muscle fasciculation but was able to drink water. The degree of ataxia worsened again, the horse was unresponsive to visual stimuli and in the end the horse fell and could not rise again. An arterial blood sample obtained when the horse was laterally recumbent revealed hypoxemia (oxygen 9.7 kPa; reference range, 9.8–14.6 kPa), hypokalemia (potassium 3.1 mmol/L; reference range, 3.3–4.5 mmol/L), hyperchloremia (chlorine 105 mmol/L) and hypoglycemia (glucose 1.1 mmol/L). Infusion of a 5% glucose solution (Fresenius Kabi AB, Uppsala, Sweden) intravenously was started, and after approximately 5 minutes the horse rose without apparent problems. The infusion of glucose 5% was continued and infusion of Ringers solution (Fresenius Kabi AB, Uppsala, Sweden) was started. In total 4 litres glucose 5% and 5 litres Ringers were administered over a period of 2 hours. The horse became responsive to the environment and muscle fasciculations were not observed following the onset infusion. Once the horse appeared stable, a venous blood sample for biochemistry and haematology was obtained. These samples identified hypoalbuminemia (albumin 25 mg/dL; reference range, 28–37 mg/dL), hyperbilirubinemia (total bilirubin 169 μmol/L; reference range, 6–32 μmol/L), hypertriglyceridemia (triglycerids 1.0 mmol/L; reference range, 0.1–0.5 mmol/L), hyperglycemia (glucose 10.4 mmol/L; reference range, 4.2–6.4 mmol/L), leukocytosis (leukocytes 13.1 × 10^9^/L; reference range, 5.5–12.0 × 10^9^/L), anaemia (erythrocytes 4.45 × 10^12^/L; reference range, 6.5–11.5 × 10^12^/L), neutrophilia (neutrophils 12.0 × 10^9^/L; reference range, 2.1–6.0 × 10^9^/L) and lymphopenia (lymphocytes 0.9 × 10^9^/L; reference range, 1.7–5.0 × 10^9^/L). A per rectum abdominal palpation was performed and neither the size nor the texture of the abdominal mass appeared to have changed. The horse had also started to pass faeces containing mineral oil. An abdominocentesis was not performed due to the risk of perforating the abdominal mass. A transabdominal ultrasound examination of the left and ventral parts of the abdomen was performed. A well demarcated large heterogeneous mass could be identified in the ventral abdomen extending to the maximum penetration of the probe, 26 cm into the abdominal cavity. The mass contained pockets of anechoic fluid, and around the mass normal intestines could easily be identified. Some segments of the small intestines showed hyperperistalsis with increased amounts of fluids within the lumen. No further abnormalities were observed. The findings were consistent with a large intra-abdominal tumour and in agreement with the owner the horse was euthanized and a necropsy performed.

### Necropsy

**Figure 1:**
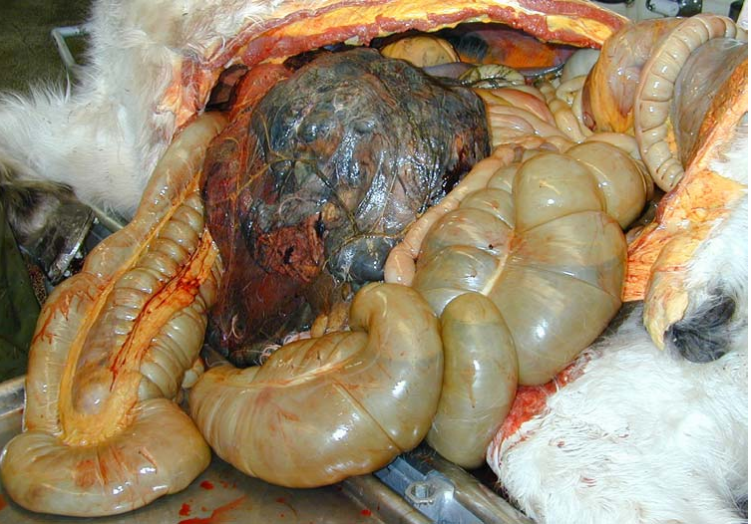
Necropsy revealed an approximately 50 × 50 × 30 cm neoplastic mass weighing 37 kg. The tumour was enclosed within the omentum majus, located between the liver and stomach cranially and the colon caudally, and attached by a relatively small stalk to the pyloric part of the stomach.

**Figure 2:**
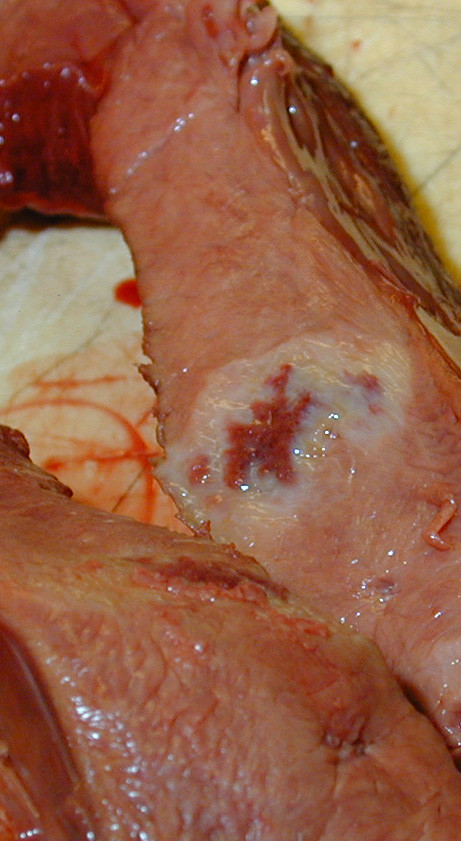
The ventricular septum of the heart contained several round nodules poorly demarcated from the surrounding myocardium.

Necropsy revealed an approximately 50 × 50 × 30 cm intraabdominal mass (Fig 1), which weighed 37 kg and was enclosed within the greater omentum, located between the liver and stomach cranially and the colon caudally, and attached by a relatively small stalk to the pyloric part of the stomach. It consisted of yellowish lobules of soft and fragile tissue divided by darker areas of haemorrhage and necrosis. Approximately 10 L of sanguineous abdominal fluid was also found. The ventricular septum of the heart contained several round nodules poorly demarcated from the surrounding myocardium (Fig 2). The nodules had a central haemorrhagic area surrounded by a pale tissue.

### Histology

Tissue samples from the heart, lungs, pancreas, liver, kidneys, gastric wall and the tumour were fixed in 10% phosphate-buffered formalin, dehydrated and embedded in paraffin. About 4 μm sections were cut and stained with hematoxylin-eosin (HE) and van Gieson (vG). Additional sections were immunostained for cytokeratin, vimentin, desmin, smooth muscle actin (SMA) and human CD117 (also called KIT) using commercially available monoclonal (cytokeratin, vimentin, desmin and SMA) and polyclonal (CD117) antibodies (Dako Cytomation, Glostrup, Denmark). The sections were pre-treated with either trypsinization (cytokeratin and vimentin) or microwaving in 0,01 M citrate at 92°C for 15 + 5 minutes (desmin, SMA) or microwaving in TRIS-EDTA (Sigma-Aldrich, Oslo, Norway) at pH 9 for 2 × 5 + 5 minutes (CD117). The sections were treated with 3% H_2_O_2 _in methanol (Chemi-Teknik A/S, Oslo, Norway) for 10 minutes to block endogenous peroxidase activity, rinsed in Tris-HCl (Sigma-Aldrich, Oslo, Norway)-buffered saline (TBS) and incubated for 20 minutes with 2% normal goat serum. The normal goat serum was tapped off and the sections were rinsed with TBS and incubated with the primary antibodies diluted in 1% bovine serum albumin in TBS for one hour at room temperature. The visualization of the antibody-antigen complex was obtained, after rinsing with TBS, by incubation with horseradish peroxidase-labelled polymer conjugated to goat anti-mouse (vimentin, cytokeratin, desmin, SMA) or goat anti-rabbit (CD117) IgG (kits from Dako Cytomation, Glostrup, Denmark) and a final incubation with ready-to-use AEC+ (3-amino-9ethylcarbazole) substrate-chromogen solution for 10 (SMA), 15 (cytokeratin, vimentin and desmin) or 20 (CD117) minutes. (kits from Dako Cytomation, Glostrup, Denmark).

**Figure 3:**
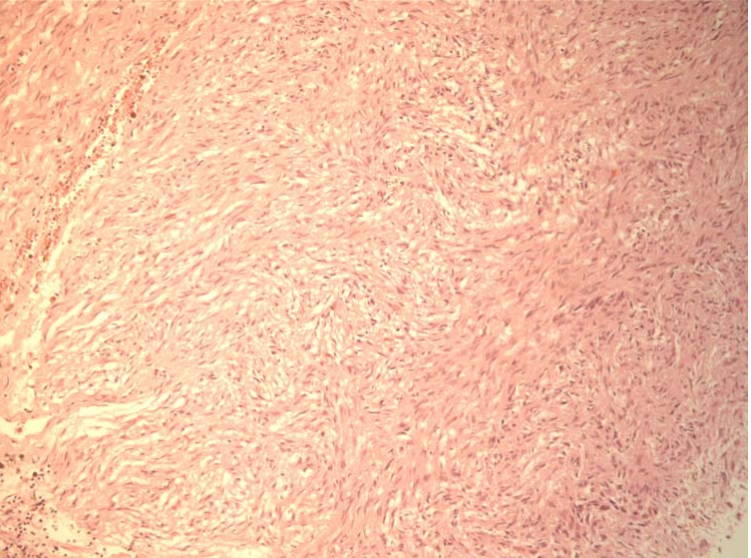
The tumour consisted of densely cellular tissue with an infiltrative growth pattern surrounded by a fibrous capsule. The cells were arranged in closely-packed streams or herring-bone patterns with sparse fibrovascular stroma, indicating a mesenchymal origin. (HE, 100×).

**Figure 4:**
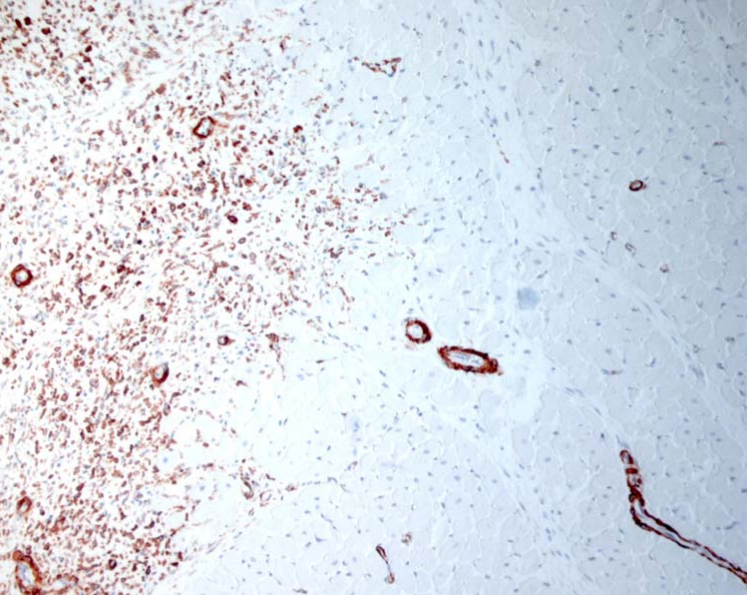
Both the tumour associated with the stomach and the nodules in the heart showed strong and diffuse positive staining for smooth muscle actin (mouse monoclonal antihuman smooth muscle actin visualized by staining with AEC (3-amino-9ethylcarbazole) labelled secondary antibody, Dako Cytomation, Glostrup, Denmark), prompting a diagnosis of a leiomyosarcoma. Here, positive neoplastic cells (red) are infiltrating (from the left to the right side) in between the negative myocardial cells. Note that also the smooth muscle cells of vessels show strong positive staining (as expected). (anti-smooth muscle actin and HE, 100×).

Histological examination demonstrated that the submucosa in the pyloric part of the stomach was dominated by large vessels with thick, but loosely woven muscular tunica media. The lumen of these vessels contained, in addition to erythrocytes, numerous elongated cells with eosinophilic cytoplasm and oval nuclei. Many of these cells seemed to be adherent to each other. The blood vessels penetrated the muscular layers of the stomach and entered the tumour. This mass consisted of densely cellular tissue with an infiltrative growth pattern surrounded by a fibrous capsule. The cells were arranged in closely-packed streams or herring-bone patterns with sparse fibrovascular stroma (Fig 3). The cells were spindle-shaped and medium-sized with indistinct cell borders and moderate amounts of eosinophilic, fibrillar cytoplasm and had an elongate, central nucleus with normochromatic, coarsely stippled chromatin. The cell population showed only moderate degree of anisocytosis and anisokaryosis and there were few mitoses. In some areas the cells were considerably thinner and separated by unstained material, giving the tissue a myxomatous character. Extensive areas throughout the tumour were dominated by haemorrhages and necrosis. The nodules in the septum of the heart consisted of similar tissue with infiltrative growth in the surrounding myocardium. The neoplastic tissue had foci of positive staining for vimentin, was negative for cytokeratin and desmin, and showed strong diffuse positive staining for SMA (Fig 4). The tumour associated with the stomach showed moderate diffuse positive staining for human CD117, while the nodules in the heart were negative for this antigen.

## Discussion

The morphological and immunohistochemical features of the tumour supported a diagnosis of gastrointestinal stromal tumour (GIST) of the leiomyosarcoma subset [8-11]. The nodules in the heart were considered as metastases from the neoplastic tissue associated with the stomach.

The horse had a history of intermittent colic, possibly due to the space occupied by the tumour and multiple adhesions to other abdominal organs. GIST and leiomyosarcomas in the horse are rare occurrences [12], but abdominal leiomyomas and leiomyosarcoma have been reported to cause colic, and successful surgical removal has been described [13].

It is reasonable to assume that the neurological clinical signs reported by the owner as well as those observed at the university clinic were caused by hypoglycemia, and the hypoglycemia may thus be termed recurrent. The described clinical signs of ataxia, confusion, blindness, excessive sweating, seizures, paresis, and muscular fasciculation have previously been observed during hypoglycemia in equines [3,14]. These signs were observed on several occasions by the owner, and one occasion in the hospital. On two separate occasions blood samples confirming hypoglycemia was obtained, and there was an almost immediate remission of clinical signs following intravenous infusion of glucose. The second hypoglycemic episode observed in the hospital may have been partially instituted by rebound hypoglycemia when intravenous glucose therapy was discontinued. However the clinical signs reported by the owner may not be attributed to rebound hypoglycemia.

Liver failure may induce hypoglycemia [14], and hyperbiliruninaemia was diagnosed in this horse. No major pathological change was found at necropsy or histopathology making it unlikely that the hyperbilirubinaemia was caused by liver damage. A more reasonable cause is the reduced food intake. A mild hypertriglyceridemia was also found indicating a catabolic state possibly caused by reduced feed intake, stress and the metabolic demand of the tumour. Hypertriglyceridemia may have contributed to the clinical signs, but was in our opinion not severe enough to explain all the clinical findings. On admission the horse had increased pulse rate and brick coloured mucous membranes, which could be consistent with endotoxaemia. Endotoxaemia may induce hyper- or hypoglycemia [15]. After fluid and flunixine meglumine therapy the horse improved clinically, but deteriorated again until the horse was recumbent. Shortly after intravenous glucose infusion the horse was able to stand again. We find it unlikely that the possible endotoxaemia was severe since profound cardiovascular compromise was not observed. The possible endotoxaemia and reduced food intake may have contributed to the hypoglycemia, but in our opinion it is unlikely that they were sufficient to cause such a severe hypoglycemia.

Hypoglycemia causing clinical signs in adult horses is a rare occurrence, but has previously been described in association with tumours. One pony had a pancreatic islet adenoma which was associated with hyperinsulinism causing hypoglycemia [3]. Hypoglycemia in horses has previously been reported in association with renal carcinoma [4,6], hepatocellular carcinoma [7] and peritoneal mesothelioma [5] without a definite cause being established. Hypoglycemia in association with tumours is well recognised in man and dogs, and GIST and leiomyosarcomas causing hypoglycemia have been described in both species [1,16]. Tumours may cause hypoglycemia by different mechanisms; functional insulinomas produce insulin and thus lower blood glucose. Possible explanations for non islet cell hypoglycemia has been proposed to be the catabolism of the tumour lowering blood glucose, secretion of substances which have insulin-like effect, interference with liver function and suppression of counterregulatory hormones [2]. In some species overt insulin-like growth factor 2 (IGF-2) is thought to cause hypoglycemia [16,17]. One case report investigated the level of IGF-2 in a horse with renal cell carcinoma and hypoglycemia, but the data were inconclusive [4]. The plasma level of IGF-2 was not measured in the current case, and the exact mechanism causing hypoglycemia was not determined.

## Conclusion

This case indicates that GIST may cause hypoglycemia in the horse.

## Competing interests

The authors declare that they have no competing interests.

## Authors' contributions

HAH carried out the clinical examination and treatment and drafted the manuscript. BY carried out the necropsy, IJR performed the immunohistochemical staining and BY did the histological examination. NO carried out the ultrasound examiniation. All authors read and approved the final manuscript.
